# Synchronization of spontaneous eyeblinks while viewing video stories

**DOI:** 10.1098/rspb.2009.0828

**Published:** 2009-07-29

**Authors:** Tamami Nakano, Yoshiharu Yamamoto, Keiichi Kitajo, Toshimitsu Takahashi, Shigeru Kitazawa

**Affiliations:** 1 Graduate School of Education, University of Tokyo, Tokyo, Japan; 2 Japan Society for the Promotion of Science, Tokyo, Japan; 3 Department of Physiology, Juntendo University School of Medicine, Tokyo, Japan; 4 CREST, Japan Science and Technology Agency, Saitama, Japan; 5 Laboratory for Dynamics of Emergent Intelligence, RIKEN Brain Science Institute, Saitama, Japan

**Keywords:** spontaneous eyeblink, synchronization, attention

## Abstract

Blinks are generally suppressed during a task that requires visual attention and tend to occur immediately before or after the task when the timing of its onset and offset are explicitly given. During the viewing of video stories, blinks are expected to occur at explicit breaks such as scene changes. However, given that the scene length is unpredictable, there should also be appropriate timing for blinking within a scene to prevent temporal loss of critical visual information. Here, we show that spontaneous blinks were highly synchronized between and within subjects when they viewed the same short video stories, but were not explicitly tied to the scene breaks. Synchronized blinks occurred during scenes that required less attention such as at the conclusion of an action, during the absence of the main character, during a long shot and during repeated presentations of a similar scene. In contrast, blink synchronization was not observed when subjects viewed a background video or when they listened to a story read aloud. The results suggest that humans share a mechanism for controlling the timing of blinks that searches for an implicit timing that is appropriate to minimize the chance of losing critical information while viewing a stream of visual events.

## 1. Introduction

Our visual world is interrupted by eyeblinks every few seconds. If we assume that 400 ms is lost per blink (VanderWerf *et al*. 2003), the loss time would amount to 6 s min^−1^ or 10 per cent of total viewing time. However, we rarely notice this profound interruption. In addition to physically blocking out visual information, blinking triggers the active suppression of information intake as much as 50 ms prior to blink initiation, an effect known as blink suppression (Volkmann *et al*. 1980; Volkmann 1986). Because the sensitivity to low spatial frequency visual stimuli is selectively suppressed during blinks, this blink suppression affects visual processing in the magnocellular pathway in a way similar to saccadic suppression (Volkmann 1986; Ridder & Tomlinson 1993; Burr *et al*. 1994; Ridder & Tomlinson 1995; Ridder & Tomlinson 1997). A recent neuroimaging study revealed that the prefrontal and parietal cortices as well as the visual cortex showed decreases in activity during blinks (Bristow *et al*. 2005*b*). The suppression of the magnocellular pathway and cerebral cortices may contribute to desensitizing the temporal change across blinks and to maintaining stable visual perception and awareness by rendering eyeblinks unnoticed. However, these blink-suppression mechanisms just cover up and never actually make up for the visual information lost during the eyeblink. Therefore, it is still necessary for us to choose appropriate timing for eyeblinks so that we may not lose important visual information.

Previous studies have reported that blink timing is related to explicit attentional breaks (Hall 1945; Drew 1951; Stern *et al*. 1984; Fogarty & Stern 1989; Orchard & Stern 1991; Fukuda 1994). During reading, spontaneous blinks are likely to occur at punctuation marks (Hall 1945). Similarly, blinks are generally suppressed during a task that demands visual attention and tend to occur immediately before and after the task when we are explicitly given the timing of its onset and offset (Drew 1951; Stern *et al*. 1984; Fogarty & Stern 1989; Fukuda 1994). These actual controls of eyeblinks in regard to explicit breaks of a task would contribute to minimizing the loss of important visual information. However, in our daily life, it is not likely that we are always given explicit breaks in a flow of visual information. This leads us to hypothesize that we possess a mechanism for controlling the timing of blinks that searches for an implicit break from visual streams to prevent temporal loss of critical visual information.

When watching video stories, for example, it is reasonable to expect that blinks are likely to occur at the explicit breaks of scenes. However, given that the scene length is unpredictable, there should also be an appropriate timing for blinking within a scene. If we possess a mechanism for controlling the timing of blinks that searches for an implicit break from visual streams, the blink would become synchronized not only at the explicit scene breaks but also at the implicit breaks. To test these predictions, we examined the spontaneous eyeblinks of 14 subjects while they viewed two silent video clips and listened to a narrative. One of the video clips was taken from the television programme ‘Mr. Bean’ (MB), a British comedy, which had an attractive story and was easy to understand without sound. The other video was taken from background videos (BGVs) of landscapes or tropical fish as a control that provided no story development, but only constantly changing scenes. The short narrative was taken from an audio book of ‘Harry Potter’ (HP) as another control that had a rich story, but that lacked any actual visual stimuli. The stimuli were chosen to test the hypothesis that eyeblinks become synchronized within and across subjects when the visual stimuli have rich and quick plot development because of the necessity of controlling each blink occurrence at an appropriate timing that would cause minimal loss of critical visual information.

## 2. Material and methods

### (a) Subjects

Eighteen healthy adults (nine male, nine female, age 22–31 years) with normal or corrected-to-normal visual acuity participated. Four subjects were excluded from the analysis because their mean blink rates fell outside the range of 1 s.d. around the mean (mean = 24.6, s.d. = 16.4): two because of excessive blink occurrence (mean blink rate per minute 64.5 and 43.9) and two because of scarce blink occurrence (6.1 and 7.5). All subjects gave written informed consent.

### (b) Experimental design and stimuli

Each subject participated in three experiments in which a video story taken from a British TV comedy (MB), a BGV and a narration of a story taken from HP were presented. The order of MB, BGV and HP experiments was counterbalanced across subjects. Two stimuli were prepared for each experiment, and one of the two was presented to each subject three times in each experiment. MB stories (MB1 and MB2) were taken from ‘The Best Bits’ in ‘Rowan Atkinson in Mr. Bean 1’ (2004, Universal Studios). In both MB1 (216 s) and MB2 (207 s), an actor drives a car in the street (‘Mr. Bean at the Dentist’) or in a parking garage (‘Mr. Bean Gets Out of a Parking Garage’). The stories were easy to understand without sound. There were 50 and 36 scene breaks in MB1 and MB2, respectively. BGVs (BGV1, 210 s; BGV2, 210 s) featured schools of tropical fish (BGV1) and landscapes surrounding the Aegean Sea (BGV2). The number of scene changes was 10 and 18, respectively. Audio stories (HP1, 205 s; HP2, 218 s) were taken from narrations of a Japanese actor reading the novel ‘Harry Potter and the Philosopher's Stone’ (ch. 1 and 3, respectively) in Japanese. MB and BGV were presented without any sound, and HP was presented with a blank screen. During HP, the subjects were instructed to freely look at the blank screen.

Each experiment started with a blank screen for 210 s, which served as a control. The test stimulus was then presented three times in repetition with a 60 s interval between presentations. Subsequently, the subjects answered six two-choice questions about the content of the stimuli. The subjects then moved on to the next experiment without any interval. The subjects were informed in advance that their eye movements would be measured while watching a video stimulus or listening to an audio story and they would answer multiple-choice questions after each experiment. They were never told that their blinking was being measured. The mean correct score to the questions was very high (96%, ranging from 83% to 100%), and the score did not differ significantly across the three types of experiments (one-way analysis of variance (ANOVA), *p* > 0.2).

Visual stimuli were presented on a liquid crystal display screen (DELL, 17 inches) in a dimly lit room, and auditory stimuli were presented via a loudspeaker system with a peak amplitude of 70 dB sound pressure level. The subjects sat on a chair in front of the screen, which was placed 1 m from the chair. The behaviour of subjects during the experiment was video recorded to check if they looked away from the screen.

### (c) Data acquisition

Vertical electrooculograms (EOGs) were recorded using Ag/AgCl electrodes (impedance <5 kΩ) attached to the left supra- and infraorbital sites. The reference electrode was placed on the left ear lobe. The EOG signals were amplified by a bioelectric amplifier (AB-621G, Nihon Kohden, Tokyo, Japan) with an AC time constant of 0.3 s, digitized online at a rate of 100 Hz and stored on a hard disk. The data were analysed offline.

### (d) Data analysis

To detect blink onset times, the EOG signals were smoothed (moving average with a 70 ms time window) and differentiated, and the differentiated signal was thresholded at 1.5 s.d. below the mean. If the sign of the differentiated EOG signal inverted rapidly from negative to positive and reached 1.5 s.d. above the mean within 100 ms, the point of the negative threshold was determined as an eyeblink onset time. This procedure yielded a series of blink onset times with 10 ms time resolution (Yuze & Tada 1994). For confirmation, we also counted the number of eyeblinks in close-up video pictures of the face of each subject, which we recorded during each experimental session. The number yielded by video counting matched with those yielded by the EOG analysis.

To examine whether blinks occurred at random or specific timings within each subject in each experiment, we compared the blink timing in one presentation (reference; e.g. the first presentation) with that in another presentation (test; e.g. the second presentation). For each eyeblink in the reference, we searched for the nearest eyeblink in the test and measured the eyeblink onset asynchrony between the two. We repeated the procedure for six combinations of presentations (first/second, first/third, second/third, second/first, third/first and third/second) and normalized the accumulated distribution of the onset asynchrony by the total number of eyeblinks. It is worth noting that a single normalized distribution of the onset asynchrony was prepared from six combinations for each subject. This normalized data (per cent proportion to total eyeblinks) followed normal distribution (Shapiro–Wilk test). In addition to this analysis in which we used the original data (OD), we repeated the same procedure after shuffling the inter-blink intervals (IBIs) of the test data (randomized data, RD). The RD preserved the exact distribution of IBIs, but lost the fine timing structure (Toyama *et al*. 1981*a*,*b*; Theiler *et al*. 1992). Thus, RD served as a control under the null hypothesis that the ‘synchronous’ eyeblinks occurred by chance. For each combination of reference and test data, we generated 40 RD from the original test data and accumulated the distribution of the onset asynchrony, which was divided by 40. As for the number of randomizations, we systematically tested with 10, 40, 100 and 1000. As a result, the number of randomizations from 10 to 1000 had little effect on the RD distribution. Thus, we adopted 40 randomizations in the present study. The procedure was repeated for six combinations of presentations, and the accumulated distribution of the onset asynchrony was normalized by the total number of eyeblinks to get one normalized blink frequency histogram for each subject. The difference between the OD and RD curves served as a measure of the net synchronization of eyeblinks. To identify the time window of the onset asynchrony showing different frequency between OD and RD, we first conducted three-way ANOVA with two within-group factors of data shuffling (OD/RD) and onset asynchrony (nine bins) and one between-group factor of stimulus (MB, MB1/MB2; BGV, BGV1/BGV2; HP, HP1/HP2). In this initial analysis, no significant main effect of the between-group factor was detected. Therefore, data were pooled over the two kinds of stimuli, and we conducted two-way ANOVA with the main effects of data shuffling (OD/RD) and onset asynchrony (nine bins) and their interaction. We repeated the whole analysis with five different bin widths (100, 200, 300, 400 and 500 ms). Because significant interaction was found with bin widths from 200 to 500 ms, we show results obtained with a bin width of 400 ms as representative data. Results for each bin width is shown in the electronic supplementary material, table S2.

We further compared raw blink counts in OD with 95th percentile thresholds calculated from 1000 RD sets. We initially looked at all bins for each subject and counted the number of subjects that exceeded the 95th percentile threshold in at least one of the nine bins, for each of MB, BGV and HP experiments. When the number of subjects exceeded a level of significance (nine subjects and above, *p* = 0.035), we further tested which bin contributed by counting the number of subjects for each bin. A bin-by-bin count was judged as significant when it was four and above (*p* = 0.038, corrected for nine repetitions).

To examine across-subject synchrony, we used data from the first presentation to each of the 14 subjects. The subjects were divided into two groups according to the stimulus (MB1/MB2, BGV1/BGV2, HP1/HP2) in each experiment. Data from one subject were used as a reference and those from another in the same group were used as a test. Thus, each subject had six combinations with the other subjects. OD and RD curves were generated in a manner similar to the within-subject analysis. The accumulated distribution of the onset asynchrony was normalized by the total number of eyeblinks in each subject. Two-way ANOVA was used to test the main effects of data shuffling (OD/RD) and onset asynchrony (nine bins) and their interaction.

To examine the relation of blink timing to scene breaks, we created a latency histogram of the first eyeblink after each scene break (see figure 3*a*, solid line, OD). By shuffling the IBIs, we generated 40 RD series and created another latency histogram that served as a control totally independent of the timing of the scene breaks (see figure 3*a*, dashed line, RD). Two-way ANOVA was used to test the main effects of data shuffling (OD/RD) and onset asynchrony (20 bins) and their interaction. Next, we examined whether the blinks following explicit scene breaks contributed to blink synchronization. For this purpose, we removed eyeblinks that followed explicit scene breaks with a latency of 400–600 ms, and tested whether the number of synchronized blinks, as represented by the difference between the OD and RD curves (OD−RD subtraction curve), decreased significantly. Two OD−RD subtraction curves were then calculated before and after the removal of blinks, and the difference between the two OD−RD subtraction curves served as a measure of the effect on the total amount of blink synchrony.

To characterize each frame of synchronous blinks, we created a blink frequency histogram for each kind of stimulus by combining blinks from 21 viewings (seven subjects × two videos × three viewings), with a bin width of 100 ms after smoothing (400 ms time window moving average). Next, we selected 17 representative synchronous eyeblinks from the frequency histogram of eyeblinks (see #1–17, blue line in figure 4*a*) by setting a peak threshold at the height of eight. Then, we asked 10 naive observers to judge whether the frames just before each synchronous eyeblink represented (i) a car without movement; (ii) a car with predictable movements; (iii) empty streets; (iv) the end of an action of a human character; (v) the end of zooming-out; (vi) a repetition of a recent scene; or (vii) anything else. The one that gained the most votes (74% on average) was considered to be the feature of the frame.

To examine whether synchronous blinks occurred after a period of blink suppression, we extracted long IBIs above 1.0 s.d. of the mean from eyeblinks in each viewing. We then created a frequency histogram of the long IBIs by combining data from 21 viewings (seven subjects × three viewings) for each stimulus. The time bin was set at 100 ms along the timeline of each stimulus.

## 3. Results

The mean blink rate across subjects during MB viewing was 16.6 min^−1^ and was significantly smaller than that during the rest state before viewing the video (24.2, *p* < 0.05, two-tailed *t*-test). Blink rates slightly increased with repetition (15.1, 16.6 and 18.2 in the first, second and third viewings, respectively), but were consistently fewer than in the rest state. In contrast, the blink rates during BGV viewing (20.0) and HP listening (26.3) were not significantly different from those during the rest state (20.8 and 22.5, respectively). The general decrease in the blink rate during MB viewing might reflect a strategy to minimize the loss of visual information (see electronic supplementary material, table S1).

**Figure 1.**
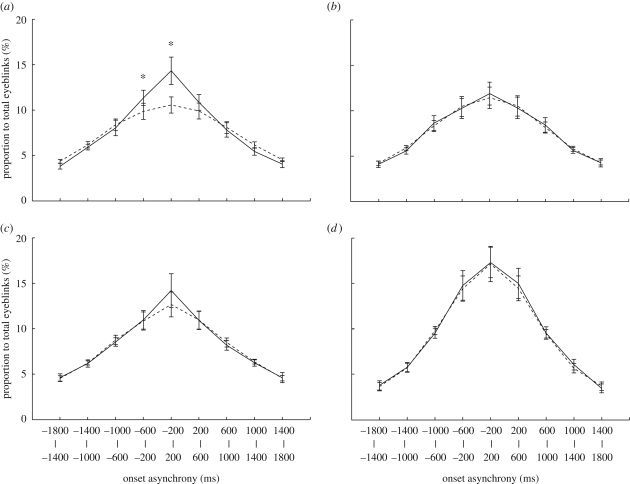
Eyeblink synchronization within subjects. (*a*), (*c*) and (*d*) Distributions of eyeblink onset asynchrony within subjects while viewing MB, viewing BGV and listening to HP, respectively. (*b*) Distribution of simulated eyeblink onset asynchrony between MB viewing and the resting state. The solid and dashed lines represent the mean distributions generated from the OD and the RD, i.e. before and after shuffling the IBIs, respectively. The difference between the two curves reflects the degree of eyeblink synchronization. Error bars indicate standard errors among subjects (*n* = 14). Asterisks indicate statistically significantly different time windows (*p* < 0.05, corrected).

Next, we examined whether blinks occurred at random or with specific timing within each subject for the three viewings of MB. The mean distribution of the onset asynchrony averaged across subjects (figure 1*a*, solid line, OD) showed a peak of 14.3 per cent in the 400 ms bin around zero, showing that 14.3 per cent of eyeblinks in one viewing was accompanied by a nearly simultaneous eyeblink in another viewing within a time window of 400 ms. But the peak synchrony dropped to 10.6 per cent after shuffling (figure 1*a*, dashed line, RD). Two-way ANOVA detected significant main effects of data shuffling (original and randomized) (*F*_(2−1),(14−1)_ = 7.0, *p* < 0.02) and onset asynchrony (nine bins) (*F*_(9−1),(9−1)*(14−1)_ = 35.6, *p* < 0.0001), and a significant interaction between data shuffling and onset asynchrony (*F*_8,104_ = 8.8, *p* < 0.0001). With regard to the interaction, there were significant simple main effects of data type around zero (−200 to 200 ms, *F*_(2−1),(2−1)*(14−1)*9_ = 59.5, *p* < 0.0001; −600 to −200 ms, *F*_1,117_ = 9.2, *p* < 0.003). To validate the shuffling method, we applied the method to eyeblinks during MB viewing (3.5 min) and those in the rest state before the session (3.5 min), which should be independent of each other. As expected, the distributions of eyeblink onset asynchrony before and after shuffling (figure 1*b*, solid and dashed lines, respectively) were almost identical. In contrast to MB viewing, the main effect of data shuffling was not significant in BGV viewing or listening to HP (figure 1*c* and *d*). Although the main effect of onset asynchrony was significant in both BGV and HP (*F*_8,104_ = 20.8, *p* < 0.0001; *F*_8,104_ = 26.5, *p* < 0.0001, respectively), there was no significant interaction. These results suggest that at least some eyeblinks occurred with specific timing while viewing MB, but not while viewing BGV or listening to HP.

We further compared each observed blink count in OD with a 95th percentile threshold defined from 1000 RD. This randomization test revealed that 10 of 14 subjects exceeded the threshold in at least one of nine bins in the MB condition (see electronic supplementary material, figure S1). The number was significant in that the probability of observing 10 or more subjects by chance is 0.0094. A *post hoc* test revealed that the number of subjects reached a level of significance in the fourth (−600 to −200 ms, *n* = 4; *p* = 0.038) and the fifth (−200 to +200 ms, *n* = 7; *p* = 0.000 018) bins. On the other hand, the same randomization test revealed that the 95 per cent criterion was satisfied in only 5 of 14 subjects in both BGV and HP conditions. The number (five and more) was as expected by chance (*p* = 0.64). These results support our conclusions that the spontaneous eyeblinks were synchronized while viewing video stories, but not in the other conditions.

**Figure 2.**
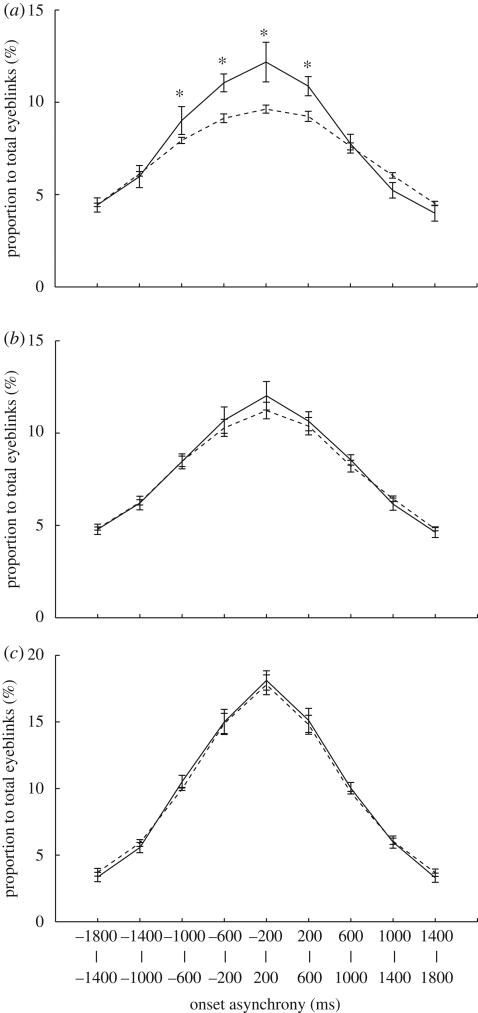
Eyeblink synchronization across subjects. Distributions of eyeblink onset asynchrony across subjects while (*a*) viewing MB, (*b*) viewing BGV and (*c*) listening to HP. Note the significant synchronization in MB viewings. Other conventions are the same as in figure 1.

Next, we examined whether blinks occurred with specific timing across subjects when each subject viewed one MB video clip for the first time. Despite the diversity of the spontaneous blink rate (electronic supplementary material, table S1), there was a prominent peak in synchronous eyeblinks (12.2%, figure 2*a*, solid line) compared with the peak with RD (9.6%). Two-way ANOVA detected significant main effects of data shuffling (*F*_1,13_ = 18.8, *p* < 0.001) and onset asynchrony (*F*_8,104_ = 56.8, *p* < 0.0001) and a significant interaction (*F*_8,104_ = 4.5, *p* < 0.0001). With regard to the interaction, there were significant simple main effects of data shuffling in four time bins around zero (−200 to 200 ms, *F*_1,117_ = 21.6, *p* < 0.0001; −600 to −200 ms, *F*_1,117_ = 11.4, *p* < 0.001; 600 to 200 ms, *F*_1,117_ = 10.3, *p* < 0.002; −1000 to −600 ms, *F*_1,117_ = 4.2, *p* < 0.04). In contrast to MB viewing, the interaction between the effects of data shuffling and onset asynchrony was not significant for either BGV viewing or listening to HP (figure 2*b*,*c*). Thus, some eyeblinks during MB viewing occurred with some specific timing shared among subjects.

**Figure 3.**
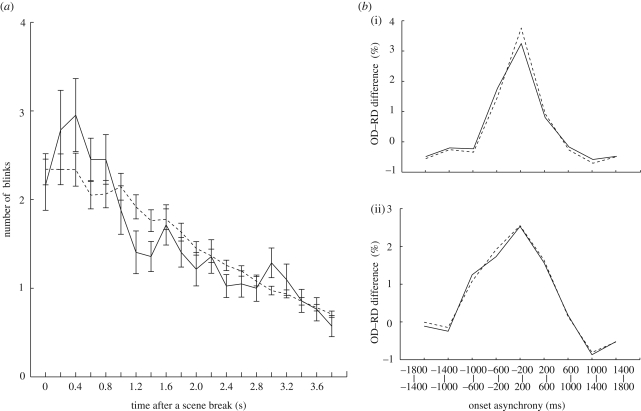
Eyeblinks after explicit scene breaks. (*a*) Onset latency histograms of the first eyeblink after a scene break in MB viewing (solid line, OD). The peak at 0.4 s disappeared after shuffling the IBIs (dashed line, RD). Error bars indicate standard errors among all trials (*n* = 42; 14 subjects × three viewings). (*b*) (i) Within subjects; (ii) across subjects. Net blink asynchrony before (dotted line) and after (solid line) removing blinks that occurred with a latency of 0.4–0.6 s. Dotted lines represent subtractions of RD curves from OD curves in the OD (figure 1*a*, within subjects; figure 2*a*, across subjects). Solid lines are data after the removal of immediate blinks after scene breaks. The removal had little effect on the total amount of blink synchrony.

It could be argued, however, that blinks that occurred repeatedly within or across subjects merely occurred in response to explicit breaks in scenes in the video stories. To test this possibility, we next examined the timing of the first eyeblink after each scene break. The rate of the first blink gradually increased and showed a peak at 400–600 ms after the scene break (figure 3*a*, solid line, OD). The peak disappeared after shuffling the order of eyeblinks (figure 3*a*, dashed line, RD). Two-way ANOVA detected a significant interaction between data shuffling and time (*F*_19,779_ = 2.2, *p* < 0.003) that was caused by the difference at the peak (OD > RD, 400–600 ms) (*F*_1,820_ = 9.4, *p* < 0.003) and a later reversal at 1200–1400 ms (RD > OD, *F*_1,820_ = 6.5, *p* < 0.02). Thus, some blinks did occur in response to explicit breaks, with a latency of 400–600 ms (post-explicit-break eyeblinks). Furthermore, we examined whether the blink synchronization could be completely explained by the post-explicit-break eyeblinks by comparing the degree of synchronization before and after removing the ones with latencies between 400 and 600 ms, following explicit scene breaks (5% of the total eyeblinks). The OD−RD difference curves showed a peak of 3.7 per cent (within-subject analysis) and 2.6 per cent (across-subject analysis) at zero time lag before the removal (figure 3*b*, dotted curves). The peak difference after removing the blinks (figure 3*b*, solid curves) remained as high (3.2% and 2.6%) as before removing the blinks. Two-way ANOVA (removal × time lag) indicated no significant main effect of the removal or interaction between the removal and time lag in both within- and across-subject analyses. By applying the same analysis to the data in the BGV condition, we found that the removal of blinks within the time window (0.4–0.8 s) after the explicit scene break had little effect: these blinks in response to the explicit scene breaks explained less than 0.1 per cent of the synchronized blinks in both within- and between-subject analyses. This indicates that the number of synchronized blinks in the BGV condition would increase by less than 0.5 per cent (0.1 × 5), even if the number of explicit scene breaks increased from 10 to 50, which is the same number of explicit scene breaks in the MB1 condition. These results suggest that the observed synchronization of blinks in the MB condition was not caused by the explicit breaks.

**Figure 4.**
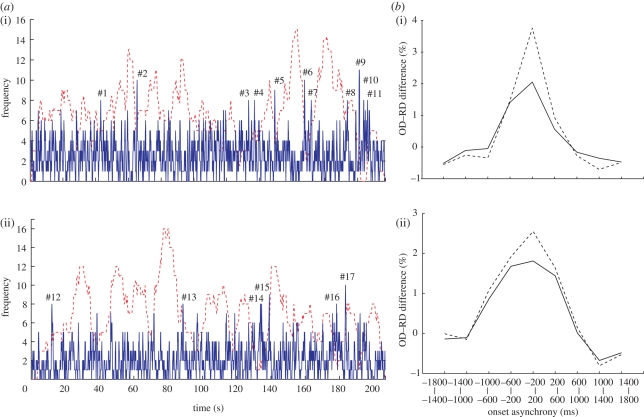
Timing of synchronous blinks. (*a*) Frequency histograms of eyeblinks (blue solid line) and long IBIs (red dotted line) along the timeline of (i) MB1 and (ii) MB2. Peaks of eyeblink frequency greater than eight are numbered from #1 to #11 (MB1) and from #12 to #17 (MB2). (*b*) Net blink asynchrony before (dotted line) and after (solid line) removing the blinks at the numbered peaks (greater than or equal to 8). Two-way ANOVA detected a significant interaction between the removal and time lag, caused by the difference at the peaks ((i) within subjects, *F*_1,117_ = 75.5, *p* < 0.0001; (ii) across subjects, *F*_1,117_ = 25.1, *p* < 0.0001).

To identify the source of blink synchrony, we created a blink frequency histogram along the timeline of each video (figure 4*a*, blue solid line, MB1 and MB2). There were some distinct peaks, as large as 11 at the maximum, in comparison with the mean frequency (MB1, mean 2.6, SD 1.7; MB2, mean 2.1, SD 1.7). When we removed the blinks greater than or equal to 8 (7% of total blinks, 11 and six peaks in MB1 and MB2, respectively; #1–17 in figure 4*a*), the peak synchronization dropped significantly by 1.7 per cent (within subjects; figure 4*b*i) and 0.8 per cent (across subjects; figure 4*b*ii). These blinks composed approximately 46 per cent (within subjects) and 31 per cent (across subjects) of the blinks that contributed to the observed synchronization. Furthermore, when we removed blinks greater than or equal to 6 in both MB1 and MB2, blink synchronization not only decreased but also disappeared. That we had to remove 57 (MB1) and 28 (MB2) peaks that amounted to 31 per cent (MB1) and 23 per cent (MB2) of total eyeblinks indicates that the synchronization phenomenon occurred at many time points distributed over the video clips. Eyeblinks that occurred at these time points were critical for the observed synchronization because synchronization remained after removing the same number of eyeblinks (31% and 23% in MB1 and MB2, respectively) that were chosen at random.

Furthermore, we examined whether these synchronous eyeblinks occurred after a period of long-term blink suppression. For this purpose, we extracted long IBIs above 1.0 s.d. of the mean in each viewing and created a frequency histogram of long IBIs along the timeline of each video (red dotted line in figure 4*a*; red lines in figure S2, electronic supplementary material). Next, we selected 17 representative synchronous eyeblinks from a frequency histogram of eyeblinks (#1–17, blue line in figure 4*a*) by setting a peak threshold at the height of eight. Then, we examined the relation between these two frequency histograms of long IBIs and eyeblinks. As a result, some synchronous blinks (#2, 6 and 13 in figures 4 and S2, electronic supplementary material) were observed just after the distinct peaks of long IBI frequency. On the contrary, others (#7, 15 and 17) occurred just before the peaks of long IBI frequency. However, many others (#3, 4, 10, 11, 12, 14 and 16) occurred when the long IBI frequency was low.

**Figure 5.**
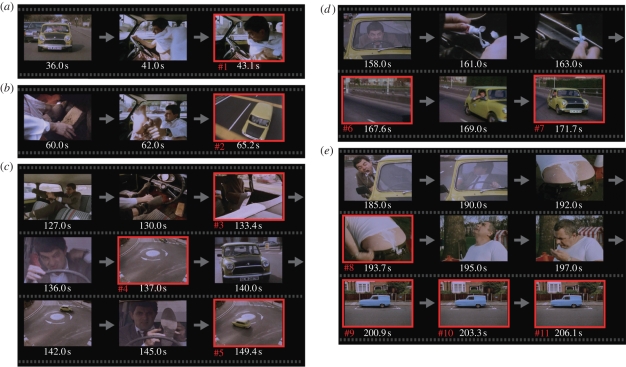
Frames of synchronous blinks (MB1). Series of video frames preceding the 11 synchronous blink onsets. The frame of the blink timing is highlighted with red and the blink number (as in figure 4) is shown below. (*a*) The main character began to put on his shirt while driving. After putting his arms through the sleeves, the blink frequency reached a high level (#1). (*b*) The main character put a brick on the accelerator pedal of the car and began to take off his pyjama trousers. After that, the blink frequency reached a high level at a distant view of his car (#2). (*c*) After he put on his socks while putting his feet on the steering wheel and then moving into the driver's seat, the blink frequency reached a high level (#3). He put a tie around his neck and put on his shoes. The blink frequency reached a high level at intervening frames of an empty crossroad (#4) and a distant view of his car (#5). (*d*) He drove his car by holding the steering wheel with his mouth and put some toothpaste on his toothbrush. The blink frequency reached a high level at intervening frames of an empty road (#6) and a distant view of his car (#7). (*e*) He spat out the windshield washer fluid with which he rinsed his mouth, and it hit another man's back, at which point the blink frequency reached a high level (#8). The man wondered if it was bird droppings, after which the blink frequency reached a high level three times during a scene with a parked car (#9–11). Movie stills courtesy of Universal Studios Licensing LLLP. *Mr. Bean Video Series.* ©Polygram Video International.

**Figure 6.**
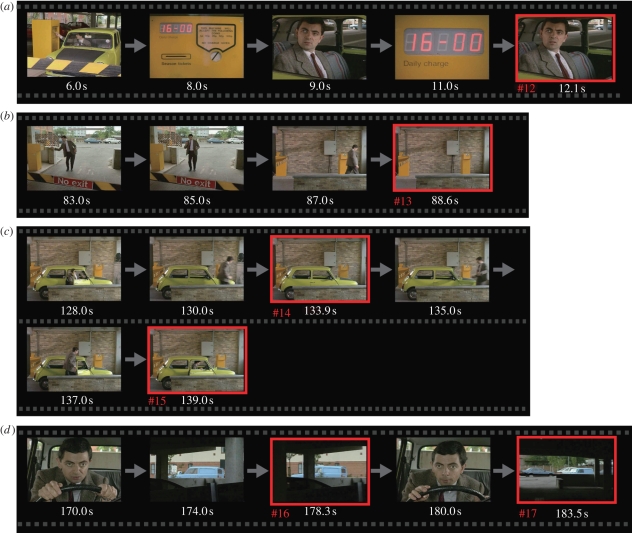
Frames of synchronous blinks (MB2). (*a*) When the main character pulled up to a tollbooth to pay the parking fee, he was surprised because the display showed ‘16.00 daily charge’. The blink frequency reached a high level at the second close-up (#12). (*b*) He tried to open the crossing bar by standing at the entrance side of the parking lot, but in vain. When he walked away from the scene, the blink frequency reached a high level (#13). (*c*) The blink frequency reached a high level when the actor got out of his car and left the scene (#14) and when he got into his car again (#15). (*d*) He waited for another car to enter the parking lot. The blink frequency reached a high level when a blue car turned the corner of the parking lot (#16) and after the scene returned to the car from a close-up scene of the main character (#17). Movie stills courtesy of Universal Studios Licensing LLLP. *Mr. Bean Video Series.* ©Polygram Video International.

Finally, we characterized frame sequences around the 17 synchronous eyeblinks in terms of the contexts of the stories (figures 5 and 6 for MB1 and MB2, respectively). In general, blinks were suppressed while an action of the main character was unfolding and were released at less animated scenes. Of the frames around the 17 distinctive peaks, 11 (64%) presented no human characters but only a car without movement (#9, 10, 11 and 14), a car with predictable movements (#2, 5, 16 and 17) and empty streets (#4, 6 and 13). Of the other six peaks, four (24%) were classified as scenes that concluded an action of a human character (#1, 3, 8 and 15). Of the remaining two peaks, one (6%) occurred near the end of a wide-angle shot (#7) and the other (6%) occurred at frames almost identical to a recent scene (#12). These observations suggest that the synchronized blinks occurred selectively at implicit breaks in the video stories as assessed unconsciously, but commonly, by the subjects.

## 4. Discussion

Spontaneous eyeblinks were synchronized both within and across subjects when they viewed the same video stories. This blink synchronization was not observed when they viewed BGVs that did not contain any story or when they listened to a narrated story. Thus, the synchronization required a story, but the need to follow a storyline *per se* was not the cause of synchronization. The blink synchrony occurred only when subjects had to follow a storyline by extracting information from a stream of visual events.

Previous studies have reported that blink timing is related to explicit attentional breaks (Hall 1945; Drew 1951; Stern *et al*. 1984; Fogarty & Stern 1989; Fukuda 1994). Thus, it is reasonable to expect that blinks are synchronized to the explicit breaks of scenes when watching video stories. As expected, the blink rate increased at 400–600 ms after scene breaks. Because the latency of 400–600 ms was much longer than that of the photic blink reflex in response to a photic stimulus (less than 100 ms) (Yates & Brown 1981; Mukuno *et al*. 1983), we consider that the blinks were not triggered reflexively by physical stimuli such as a rapid change in luminance at the scene change, but were selectively generated as a result of cognitive processing triggered by the explicit break.

The blinks immediately after the explicit breaks, however, explained only a small non-significant part of the total blinks that occurred in synchrony within and across subjects (figure 3*b*). After excluding the blinks that occurred in response to the explicit breaks, we still observed as much blink synchronization as before these blinks were excluded, suggesting that blinks following explicit scene breaks did not comprise a significant component of blinks synchronization. We do sometimes blink following explicit scene breaks, but maybe at random after different scene breaks. Given that scene length is unpredictable when video streams are viewed, the blink synchronization suggests that the brain searches for an implicit timing within the same scene, rather than an explicit timing, for blink generation.

However, it may be argued that synchronous blinks were caused by a scene-dependent mechanism for blink suppression coupled with a mechanism for spontaneous blink generation that is not scene dependent. By examining the relation between the synchronous blinks and the long IBIs, some synchronous blinks evidently occurred just after a long period of blink suppression. However, many other synchronous blinks occurred independently of or even before long blink suppression. This result suggests that we actively search for the implicit timing which is appropriate for the blink generation while watching video streams. In fact, synchronous blinks selectively occurred at frames that seemed to be of lesser importance such as at the conclusion of an action, at the disappearance of the main character, during a long shot and during repeated presentations of a similar scene. These frames might be implicit breaks in the video stories that were detected in common within and across subjects. Furthermore, given that cortical activity during movie viewing is highly synchronized between individuals (Hasson *et al*. 2004), humans could share similarities in recognizing visual information and assessing the optimal breaks within it.

To generate a blink at such an implicit break, it is necessary both to suppress a reflexive blink and to send a controlling signal for activating lower-level blink generation mechanisms at an appropriate timing. The neural circuit of blink generation is tonically suppressed by the superior colliculus (SC) (Basso & Evinger 1996; Basso *et al*. 1996), which is under the control of the striatum and higher cortical areas such as the supplemental eye field, frontal eye field, posterior parietal cortex (PPC) and occipital cortex. These cortical areas are activated in relation to the control of voluntary and/or spontaneous eyeblinks (Bodis-Wollner *et al*. 1999; Kato & Miyauchi 2003; Bristow *et al*. 2005*a*,*b*; Yoon *et al*. 2005). Whereas these regions are also involved in the control of eye movement, the activation of the PPC during blinks is greater than during saccades (Bodis-Wollner *et al*. 1999), and the microstimulation of area 7a in monkey, a homologue of the human PPC, constantly elicits blinks during fixation (Shibutani *et al*. 1984). The PPC is also involved in sensory-motor transformation and attentional processing (Andersen *et al*. 1997), especially in the process of disengagement of covert visual attention (Posner *et al*. 1984). These previous findings suggest that the neural networks involving the PPC may play critical roles in controlling an eyeblink at implicit breaks in video stories.

Not only blinks but also saccades render us blind at two or three per second, although the physical degradation of visual input produced by a saccade typically is considerably shorter than that produced by a blink. In addition, a saccade is often accompanied by a blink (Fogarty & Stern 1989). Both involve activations in the same cortical areas (Bodis-Wollner *et al*. 1999). These facts suggest that blinks and saccades are not independent, and saccades are also generated at or near the implicit breaks in video stories. This point requires future investigations.

Despite the diversity of spontaneous blink rates and blink patterns among individuals (Ponder & Kennedy 1927), we found the synchronization of blink timing within and across individuals at implicit breaks in video stories. Our results suggest that humans share a mechanism for controlling the timing of blinks that searches for the appropriate timing to prevent the loss of critical information from the flow of visual information. This excellent control of blink generation may be closely related to the visual attentional system and contributes to stable visual perception and awareness across the interruptions of blinks.

## References

[RSPB20090828C1] Andersen R. A. Snyder L. H. Bradley D. C. Xing J. 1997 Multimodal representation of space in the posterior parietal cortex and its use in planning movements. Annu. Rev. Neurosci. 20, 303–330 (doi:10.1146/annurev.neuro.20.1.303) 9056716 10.1146/annurev.neuro.20.1.303

[RSPB20090828C2] Basso M. A. Evinger C. 1996 An explanation for reflex blink hyperexcitability in Parkinson's disease. II. Nucleus raphe magnus. J. Neurosci. 16, 7318–7330 8929438 10.1523/JNEUROSCI.16-22-07318.1996 PMC6578942

[RSPB20090828C3] Basso M. A. Powers A. S. Evinger C. 1996 An explanation for reflex blink hyperexcitability in Parkinson's disease. I. Superior colliculus. J. Neurosci. 16, 7308–7317 8929437 10.1523/JNEUROSCI.16-22-07308.1996 PMC6578952

[RSPB20090828C4] Bodis-Wollner I. Bucher S. F. Seelos K. C. 1999 Cortical activation patterns during voluntary blinks and voluntary saccades. Neurology 53, 1800–1805 10563631 10.1212/wnl.53.8.1800

[RSPB20090828C5] Bristow D. Frith C. Rees G. 2005a Two distinct neural effects of blinking on human visual processing. Neuroimage 27, 136–145 (doi:10.1016/j.neuroimage.2005.03.037) 15893941 10.1016/j.neuroimage.2005.03.037

[RSPB20090828C6] Bristow D. Haynes J. D. Sylvester R. Frith C. D. Rees G. 2005b Blinking suppresses the neural response to unchanging retinal stimulation. Curr. Biol. 15, 1296–1300 (doi:10.1016/j.cub.2005.06.025) 16051173 10.1016/j.cub.2005.06.025

[RSPB20090828C7] Burr D. C. Morrone M. C. Ross J. 1994 Selective suppression of the magnocellular visual pathway during saccadic eye movements. Nature 371, 511–513 (doi:10.1038/371511a0) 7935763 10.1038/371511a0

[RSPB20090828C8] Drew G. 1951 Variations in reflex blink-rate during visual-motor tasks. Q. J. Exp. Psychol. 3, 73–88 (doi:10.1080/17470215108416776)

[RSPB20090828C9] Fogarty C. Stern J. A. 1989 Eye movements and blinks: their relationship to higher cognitive processes. Int. J. Psychophysiol. 8, 35–42 (doi:10.1016/0167-8760(89)90017-2) 2584081 10.1016/0167-8760(89)90017-2

[RSPB20090828C10] Fukuda K. 1994 Analysis of eyeblink activity during discriminative tasks. Percept. Mot. Skills. 79, 1599–1608 7870552 10.2466/pms.1994.79.3f.1599

[RSPB20090828C11] Hall A. 1945 The origin and purposes of blinking. Br. J. Opthalmol. 29, 445–467 (doi:10.1136/bjo.29.9.445) 10.1136/bjo.29.9.445 PMC510520 18170143

[RSPB20090828C12] Hasson U. Nir Y. Levy I. Fuhrmann G. Malach R. 2004 Intersubject synchronization of cortical activity during natural vision. Science 303, 1634–1640 (doi:10.1126/science.1089506) 15016991 10.1126/science.1089506

[RSPB20090828C13] Kato M. Miyauchi S. 2003 Functional MRI of brain activation evoked by intentional eye blinking. Neuroimage 18, 749–759 (doi:10.1016/S1053-8119(03)00005-3) 12667852 10.1016/s1053-8119(03)00005-3

[RSPB20090828C14] Mukuno K. Aoki S. Ishikawa S. Tachibana S. Harada H. Hozumi G. Saito E. 1983 Three types of blink reflex evoked by supraorbital nerve, light flash and corneal stimulations. Jpn. J. Ophthalmol. 27, 261–270 6855018

[RSPB20090828C15] Orchard L. N. Stern J. A. 1991 Blinks as an index of cognitive activity during reading. Integr. Physiol. Behav. Sci. 26, 108–116 (doi:10.1007/BF02691032) 1878317 10.1007/BF02691032

[RSPB20090828C16] Ponder E. Kennedy W. P. 1927 On the act of blinking. Q. J. Exp. Physiol. 18, 89–110

[RSPB20090828C17] Posner M. I. Walker J. A. Friedrich F. J. Rafal R. D. 1984 Effects of parietal injury on covert orienting of attention. J. Neurosci. 4, 1863–1874 6737043 10.1523/JNEUROSCI.04-07-01863.1984 PMC6564871

[RSPB20090828C18] Ridder W. H. III Tomlinson A. 1993 Suppression of contrast sensitivity during eyelid blinks. Vision Res. 33, 1795–1802 (doi:10.1016/0042-6989(93)90170-2) 8266635 10.1016/0042-6989(93)90170-2

[RSPB20090828C19] Ridder W. H. III Tomlinson A. 1995 Spectral characteristics of blink suppression in normal observers. Vision Res. 35, 2569–2578 (doi:10.1016/0042-6989(95)00011-N) 7483301 10.1016/0042-6989(95)00011-n

[RSPB20090828C20] Ridder W. H. III Tomlinson A. 1997 A comparison of saccadic and blink suppression in normal observers. Vision Res. 37, 3171–3179 (doi:10.1016/S0042-6989(97)00110-7) 9463698 10.1016/s0042-6989(97)00110-7

[RSPB20090828C21] Shibutani H. Sakata H. Hyvarinen J. 1984 Saccade and blinking evoked by microstimulation of the posterior parietal association cortex of the monkey. Exp. Brain Res. 55, 1–8 (doi:10.1007/BF00240493) 6745342 10.1007/BF00240493

[RSPB20090828C22] Stern J. A. Walrath L. C. Goldstein R. 1984 The endogenous eyeblink. Psychophysiology 21, 22–33 (doi:10.1111/j.1469-8986.1984.tb02312.x) 6701241 10.1111/j.1469-8986.1984.tb02312.x

[RSPB20090828C23] Theiler J. Eubank S. Longtin A. Galdrikian B. Farmer D. 1992 Testing for nonlinearity in time series: the method of surrogate data. Physica D 58, 77–94 (doi:10.1016/0167-2789(92)90102-S)

[RSPB20090828C24] Toyama K. Kimura M. Tanaka K. 1981a Cross-correlation analysis of interneuronal connectivity in cat visual cortex. J. Neurophysiol. 46, 191–201 6267211 10.1152/jn.1981.46.2.191

[RSPB20090828C25] Toyama K. Kimura M. Tanaka K. 1981b Organization of cat visual cortex as investigated by cross-correlation technique. J. Neurophysiol. 46, 202–214 6267212 10.1152/jn.1981.46.2.202

[RSPB20090828C26] VanderWerf F. Brassinga P. Reits D. Aramideh M. Ongerboer de Visser B. 2003 Eyelid movements: behavioral studies of blinking in humans under different stimulus conditions. J. Neurophysiol. 89, 2784–2796 (doi:10.1152/jn.00557.2002) 12612018 10.1152/jn.00557.2002

[RSPB20090828C27] Volkmann F. C. 1986 Human visual suppression. Vision Res. 26, 1401–1416 (doi:10.1016/0042-6989(86)90164-1) 3303665 10.1016/0042-6989(86)90164-1

[RSPB20090828C28] Volkmann F. C. Riggs L. A. Moore R. K. 1980 Eyeblinks and visual suppression. Science 207, 900–902 (doi:10.1126/science.7355270) 7355270 10.1126/science.7355270

[RSPB20090828C29] Yates S. K. Brown W. F. 1981 Light-stimulus-evoked blink reflex: methods, normal values, relation to other blink reflexes, and observations in multiple sclerosis. Neurology 31, 272–281 7193820 10.1212/wnl.31.3.272

[RSPB20090828C30] Yoon H. W. Chung J. Y. Song M. S. Park H. 2005 Neural correlates of eye blinking; improved by simultaneous fMRI and EOG measurement. Neurosci. Lett. 381, 26–30 (doi:10.1016/j.neulet.2005.01.077) 15882784 10.1016/j.neulet.2005.01.077

[RSPB20090828C31] Yuze H. Tada H. 1994 A computerized identification and date analysis of eyeblink EOG waves. Jpn. J. Ergon. 30, 331–337

